# The Efficient and Easy Micropropagation Protocol of *Phyllanthus niruri*

**DOI:** 10.3390/plants10102141

**Published:** 2021-10-09

**Authors:** Azal Anis Suraya, Azizah Misran, Mansor Hakiman

**Affiliations:** 1 Department of Crop Science, Faculty of Agriculture, Universiti Putra Malaysia (UPM), Serdang 43400, Selangor, Malaysia; anishinee@gmail.com (A.A.S.); azizahm@upm.edu.my (A.M.); 2 Laboratory of Sustainable Resources Management, Institute of Tropical Forestry and Forest Products, Universiti Putra Malaysia (UPM), Serdang 43400, Selangor, Malaysia

**Keywords:** *Phyllanthus niruri*, basal medium, medium strength, plant growth regulators, surface sterilization, shoot multiplication

## Abstract

*Phyllanthus niruri* (*P. niruri*) or Dukung Anak is a herbal plant in the Phyllanthaceae family that has been used traditionally to treat various ailments such as diabetes, jaundice, flu and cough. *P. niruri* contains numerous medicinal benefits such as anti-tumor and anti-carcinogenic properties and a remedy for hepatitis B viral infection. Due to its beneficial properties, *P. niruri* is overharvested and wild plants become scarce. This study was conducted to develop an appropriate in vitro culture protocol for the mass production of *P. niruri*. An aseptic culture of *P. niruri* was established followed by multiplication of explants using different types of basal medium and its strength and plant growth regulators manipulation. This study also established the induction of in vitro rooting utilizing various types and concentrations of auxin. Treatment of Clorox^®^ with 30% concentration showed the lowest percentage (%) of contamination, 4.44% in *P. niruri* culture. Nodal segments of *P. niruri* were successfully induced in full-strength of Murashige and Skoog (MS) basal media with 2.33 number of shoots, 3.11 cm length of shoot and 27.91 number of leaves. In addition, explants in full-strength MS media without any additional cytokinin were recorded as the optimum results for all parameters including the number of shoots (5.0 shoots), the length of shoots (3.68 cm) and the number of leaves (27.33 leaves). Treatment of 2.5 µM indole-3-butyric acid (IBA) showed the highest number of roots (17.92 roots) and root length (1.29 cm). Rooted explants were transferred for acclimatization, and the plantlet showed over 80% of survival rate. In conclusion, plantlets of *P. niruri* were successfully induced and multiplied via in vitro culture, which could be a step closer to its commercialization.

## 1. Introduction

Over the past several years, medicinal plants have gained greater attention and recognition in Malaysia, particularly as demand for alternative medicine and natural health products increases in 2019 [1,2]. A total of 351 hectares of Malaysian land has yielded 1317 metric tonnes of herbs in 2007. Since then, herb production grows to 2800 metric tonnes from 578 hectares of land cultivated with different herb species [3]. In addition, the Malaysia Economic Transformation Program, through the National Key Economic Areas for the agriculture sector, has identified numerous high potential herbs that can be commercially utilized as a new economic growth source in the herbal industry. One of these herbs is *Phyllanthus niruri* (*P. niruri*), identified locally as Dukung Anak [4].

*P. niruri* (Phyllanthaceae) has traditionally been used to treat several ailments such as jaundice, kidney stone, flu, fever, and diabetes [5,6,7]. Several researchers discovered that *P. niruri* possesses anti-tumor, anti-oxidant, anti-carcinogenic, and hepatoprotective properties [8,9,10]. In addition, certain phenolic compounds such as gallic acid, epicatechin, gallocatechin, epigallocatechin, epicatechin 3-O-gallate, epigallocatechin 3-O-gallate have gained significant interest from the researcher [11]. Furthermore, a novel compound named niruriside was isolated from methanol extract of *P. niruri* that can inhibit the binding of human immunodeficiency virus Rev protein (HIV REV) [10]. These secondary metabolites have a wide range of therapeutic activities that have essential pharmacological effects on humans [12]. Hence, many products were made from this medicinal plant have been successfully commercialized.

The main issue for the commercialization of herbal-based products is the uniformity and consistency of the planting materials [13]. In addition, the growing demand for medicinal plants would undoubtedly decrease the sustainable supply of raw materials in the future [14]. Overharvesting and unsustainable agriculture practices lead to many consequences, such as a limited supply of herbs [15]. Moreover, to extract elevated amounts of secondary metabolites, large quantities of raw materials are needed [16].

Plant micropropagation may serve as an alternative solution to ensure the sustainable supply of plant materials. It is an excellent technique to produce plants in a large amount in a short time [17]. Micropropagation is the in vitro aseptic culture of cells, tissues, or even organs of plants grown under a controlled environment and nutrient media for growth and multiplication. This technology can be used to eliminate diseases, for secondary metabolite production, plant improvement, and conservation of threatened and endangered species [18,19,20,21]. A study conducted by Padma and Ilyas [22] resulted in a maximum number of *P. niruri* shoots (15.28 ± 0.96) on MS medium supplemented with BAP (0.5 mg/L). In addition, a maximum number of *P. niruri* shoots (3.16 ± 0.16) was achieved from nodal explants inoculated on MS medium supplemented with BAP (3.0 mg/L) [23].

Appropriate selection of chemical sterilization is essential in the first stage of plant micropropagation. All microorganisms that potentially could contaminate the culture should be removed and eliminated [24]. This step will establish an aseptic culture to be carried out in the following phase of micropropagation. On the other hand, numerous factors affect the explant’s development in the growth and multiplication phase [25,26]. For instance, the source of plant material, kind of explant, the type of basal media and its strength and plant growth regulators and its concentration [27].

Culture medium supplemented with plant growth regulators; cytokinin and auxin were used in many plants to propagate via in vitro techniques [28,29]. Auxin and cytokinin play a crucial role in many aspects of plant development and growth [30]. The interplay of auxin and cytokinin is particularly critical for controlling a few developmental processes, such as the production and maintenance of meristems, which are necessary for the establishment of the entire plant body [31]. Cytokinins play a central role in the regeneration of multiple shoots in many medicinal plant species [32,33] and sometimes in combination with auxins. Cytokinins trigger cell division and influence differentiation [34], while auxins are a major signal for apical dominance [35] and are responsible for elongation in phototropism and gravitropism [36]. In vitro plantlets will then be transferred to potting media during acclimatization, the final stage. In this stage, the survival rate of plantlets is also influenced by the type of potting media [37].

Due to its high medicinal values and properties, *P. niruri* has captured people’s attention worldwide. The plant was harvested with no sanitary consideration taken on the collected samples. The explant obtained from wild or greenhouse is typically contaminated with microorganisms such as bacteria and fungi [38]. In vitro contamination of plant cultures could be induced by either internal explant tissue or the presence of microorganisms on the surface of the explant [39]. The microorganism would kill plants eventually, whether because of their overgrowth or the release of toxic substances into the basal medium. Hence, in the surface sterilization stage, the effectiveness of removing all microorganisms with minimal damage to the plant cell is essential. Chemical sterilants could be used to reduce the microbial contaminant and increase the percentage of survival at the same time [40].

Besides, there is no information on the production of *P. niruri* in Malaysia, suggesting that the plant has not been thoroughly utilized for commercialization, especially in Malaysia. Thus, this study may propagate *P. niruri* rapidly and serve as a preliminary analysis for future studies. The objectives of this study were to examine the effect of different types and concentrations of chemical sterilants on surface sterilization and examine the growth responses of *P. niruri* explants to different basal media and their strength and plant growth regulators.

## 2. Results

### 2.1. Surface Sterilization

Nodal segments of *P. niruri* were cultured on MS medium without any plant growth regulators following the surface sterilization procedure. There were significant differences between different chemical sterilants and their concentrations on the percentage of contamination of explant of *P. niruri*. Different concentrations of nanosilver showed no significant effect on their percentage of contamination (Table 1). For Clorox^®^, there was no significant difference between 10% and 20% Clorox^®^ on their contamination percentage. The contamination percentage on explants had no significant difference between 20% and 30% Clorox^®^. However, 10% and 30% Clorox^®^ showed a significant difference in their percentage of contamination, whereby 10% Clorox^®^ showed 130% higher contamination than 30% Clorox^®^.

### 2.2. Shoot Multiplication Using Different Basal Medium and Its Strength

The ANOVA results showed significant differences among different basal media treatments and their strength. However, results from LSD revealed that different strengths of both Murashige and Skoog (MS) and Gamborg B5 (B5) basal media markedly influent the growth responses of P. niruri explant. This can be seen in Table 2 that the increment of media strength from half- to full-strength of MS media, explants produced a positive rise in the number of shoots, length of shoot, and the number of leaves, even only a slight increment. Nevertheless, all numbers from both treatments were recorded to have no significant difference from each other.

Similarly, explants in half-strength B5 basal media generated the number of shoots, length of shoot, and the number of leaves (3.67, 3.52 cm, and 24.25, respectively) that were not significantly different from those explants developed in full strength B5 basal media treatment (3.00, 4.84 cm and 35.94, respectively).

In contrast, all parameters in higher strength of MS basal media (double strength) recorded a significantly lower number of shoots (0.67), shorter length of the shoots (1.5 cm), and fewer number of leaves (4.88) compared to the full strength of MS basal media. The increasing strength of B5 basal media, from full- to double-strength, also reduced the number of shoots with their respective values of 3.00 and 1.67. Likewise, the length of shoot and number of leaves also lessen significantly when the media strength was amplified to double strength (3.58 cm and 22.83, respectively).

### 2.3. Shoot Multiplication Using Different Plant Growth Regulators and Its Concentrations

**Figure 1:**
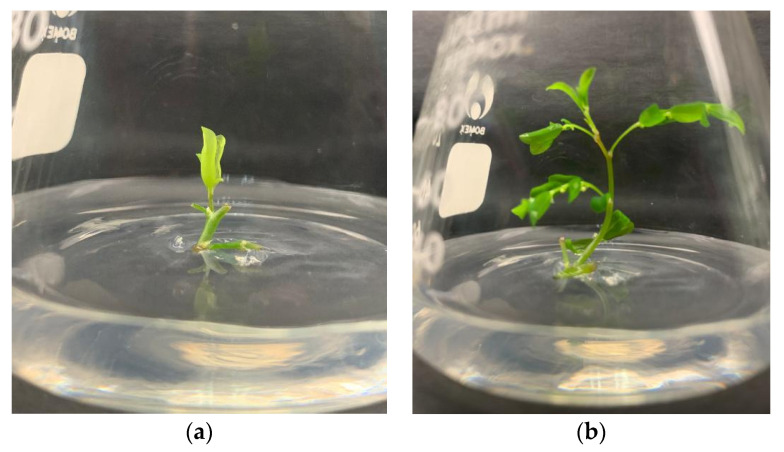
(**a**) Axillary bud proliferation of *P. niruri* in MS media; (**b**) Elongated shoot of *P. niruri* in MS media and control treatment after 2 weeks of inoculation.

The nodal segment of P. niruri showed a great response in media supplemented with cytokinin along with control treatment. The inoculated P. niruri in MS media with cytokinin treatment showed signs of axillary buds’ proliferation (Figure 1a) as early as 5 days, followed by control treatment at days six to seven. Eventually, the shoots began to develop and multiply (Figure 1b). The effect of different types and concentrations of cytokinin on the number of shoots, length of the shoot (cm), and the number of leaves were shown in Table 3.

Results in Table 3 showed P. niruri nodes maintained on medium containing 2.5 µM kinetin (Kn) and 5.0 µM 6-benzylaminopurine (BAP) showed the highest average number of shoots (5.33 shoots). However, it has no significant difference with most of the treatments including control. A similar trend also can be seen in another two parameters, length of shoots and number of leaves. On the other hand, 10.0 µM BAP generated the least number and length of shoot, 2.55 shoots, and 1.67 cm, respectively. The control treatment produced the highest number of leaves (27.33 leaves), whereas 5.0 µM 2-isopentenyl adenine (2iP) treatment showed the lowest number of leaves. In this experiment, control treatment exhibited the best result in growth parameters recorded.

### 2.4. In Vitro Rooting and Acclimatization

**Figure 2:**
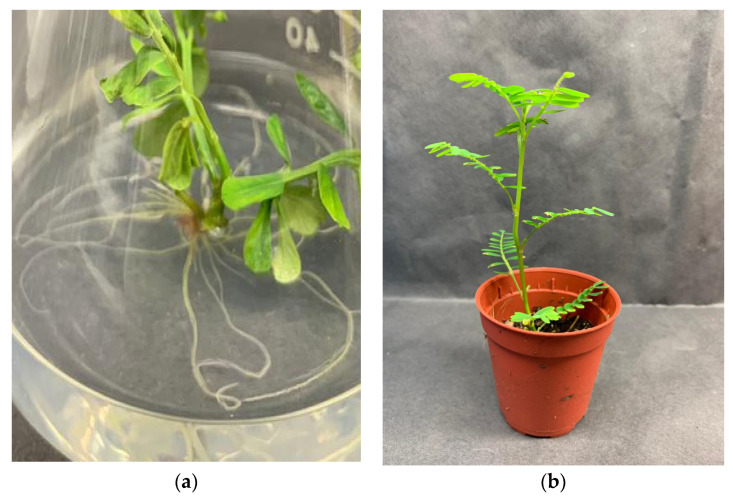
(**a**) Root development of *P. niruri* in media containing 2.5 µM IBA on week 4 after inoculation; (**b**) Survived *P. niruri* planlet in mixture of coco peat + peat moss (1:1) treatment on week 4 of acclimatization.

The media containing auxins induced a higher number of roots than the control treatment. Table 4 showed that 2.5 µM of indole-3-butyric acid (IBA) significantly affected the number of roots by producing the highest number of roots (17.92 roots) while having the longest roots (1.29 cm) (Figure 2a). However, there was no significant difference between 1.25 and 2.5 µM of IBA on the length of the root. On the other hand, there was a significant difference between all auxin treatments compared to the control treatment, which generated the least number of roots (1.0 root) and length of root (0.13 cm).

In vitro regenerated plantlets adapted well to acclimatization as plantlet leaves grow more prominent and thicken with new leaves emergence from day 8–15 (Figure 2b). Potting media of coco peat + peat moss at a ratio of 1:1 was suitable for acclimatization with 88% of plantlets survived in ex vitro condition (data not shown).

## 3. Discussion

Preventing fungal and bacterial contamination is very critical to ensure the success of micropropagation. The least percentage of contamination of explants indicated the chemical sterilants’ efficiency to remove the microorganism and establish a “clean” culture. Clorox^®^, a conventional bleach that consists of 5.25% of sodium hypochlorite (NaOCl), has the ability to penetrate the inner layers of the plant. It has been extensively used along with ethanol as an effective surface sterilizing agent in various plant species such as *Ludisia discolour* [41], *Gerbera hybrida* [42], *Ananas comosus* [43], *Jatropha curcas* [44], *Aquilaria malaccensis* [45], sour cherry [46] and *Taraxacum belorussicum* [47].

Treatment of 30% Clorox^®^ was chosen as the best surface sterilization treatment since it had the least contamination percentage. Admitting a higher concentration of bleach might diminish the contaminants, but it might induce tissue damage in the meantime [48]. The aseptic culture of *Phyllanthus caroliniensis* was established in a study conducted by Catapan et al. [49] using 2.5% active chlorine solution.

However, working with another variety, Ana et al. [24] indicated that, contamination rate decreased as active chlorine concentration increased. This supported the finding that the effect of NaOCl sterilization is associated with the chlorine ions, which generate oxidative reactions that are responsible for enzymatic inactivation and fatty acid and lipid degradation, thus, its biocide properties [50].

Nano silver was found to be an ineffective sterilant in this study. This was expected as all concentrations of nano silver gave no significant difference in the result. However, the findings differed with Rostami and Shahsavar [51], who suggested using a low concentration of nano silver as a sterilizing agent on olive explant to control the incident of microbial contaminations. This is because a high concentration of nano silver can cause severe injuries and browning of olive Mission explants. In another study, increasing the concentration of nano silver from 20–60 ppm has improved *Ocimum basilicum* L. seed yield [52]. The study conducted by Cuba-Diaz et al. [53] however showed that the silver nanoparticles (AgNO_3_) caused 100% oxidation of *Colobanthus quitensis* explants. In addition, a higher concentration of AgNO_3_ did not prevent the appearance of contaminants while a lower concentration did not promote tissue proliferation [53]. This is due to the fact that the disinfectant characteristic of AgNO_3_ is varied in its shapes and sizes [54]. Thus, the high percentages of contamination of explants in the present study are explained by the different sizes of nano silver particles and contaminants. However, the use of nanosilver solution, with sterile distilled water (SDW), resulted in no tissue injury [55].

Consequently, the full strength of MS medium (control) in this current study was found to be the most effective basal medium for the development of *P. niruri* explant as it displayed the best result in all parameters. Meanwhile, double-strength medium, regardless of the type of basal medium; MS or B5, produced the lowest results in all growth response parameters. This finding indicated that the increment of nutrient concentration inhibits the growth responses of *P. niruri*. This study’s results were in agreement with Rezali et al. [56], who stated that increased MS strength resulted in a decrease in the number and height of shoots of *Typhonium flagelliforme.* These present results are equivalent to the findings reported by Catapan et al. [57], who proved that MS media promoted a significant increase in the number of shoots and nodes generated per explant of *Phyllanthus urinaria*. MS medium was also shown to be the most suitable basal medium for the development of in vitro tubers of *Gloriosa superba,* while the B5 medium was not at all effective in induction of secondary tubers in vitro [58]. This differed from findings from a study conducted by Sadik et al. [59], who proved that B5 salts formulation had the lowest total nitrogen content, which induced stress during embryo formation of banana cultures.

Various responses from explant in producing in vitro explant on the above-mentioned basal media can be due to changes in the basal salt formulation. Nitrogen is an important component in the mineral salt formulation and it can be found in the forms of nitrate (NO_3_^−^) and ammonium (NH_4_^+^). MS medium contains two nitrogen salts, ammonium nitrate (~1650 mg/L) and potassium nitrate (~1990 mg/L), for a total nitrogen content of ~3550 mg/L [60]. This is considered to be an appropriate high nitrogen content for many plant species. Plant development is inhibited and deformed as a result of nitrogen deficiency. The most effective MS medium was found to be MS medium with a standard amount of nitrogen source in micropropagation of *Phyllanthus amarus* [61].

In the meantime, the double-strength medium’s reducing growth responses may attribute to an excessive amount of nutrients supplied. Monfort et al. [62] stated that the proper equilibrium of salts in the basal medium is crucial for explant nutrition, where double MS salt concentration inhibited growth by causing nutrient toxicity to the explant. However, a contradiction detected in a study using double salt concentration positively affects the growth of *Salicornia brachiata*, as reported by Singh et al. [63].

The number of leaves is another important parameter to consider. This is because, for certain plant species, higher shoot numbers and length of shoot do not correspond to higher leaves numbers [64]. Based on the result obtained, there is no significant difference between all treatments on the number of leaves produced except for MS double strength media which produced the least number of leaves of *P. niruri* (4.88 leaves). A similar pattern was observed in MS double strength treatment where there was a decrement in the number of shoots and length of shoot of *P. niruri*. This proven that the double-strength of MS basal media inhibited the growth and development of in vitro *P. niruri.*

This showed that the *P. niruri* explants can regenerate and produce shoots rapidly on their own, without any additional cytokinin. Presumably, in tissues that can grow without cytokinin being added (exogenous) to the medium, the cells can produce sufficient natural (endogenous) cytokinin for cell division to proceed.

This result differed with the finding by Liang and Keng [65], who stated that aseptic nodal segments of *P. niruri* cultured on MS medium fortified with 1.0 mg/L BAP produced the most number of shoots (6.6 shoots) and a combination of Kn and BAP induced multiple shoot formation in all nodal segments. Rajasubramaniam and Saradhi [66] also proved that BAP was effective for shoot multiplication of *Phyllanthus fraternus*. The application of cytokinin is essential in single node culture, according to Gallavotti [67], as it helps break the bud dormancy phase of the explants.

The endogenous amount of cytokinin present within the explant may be sufficient to induce positive organogenesis. The present study’s findings are in line with Zazimalova et al. [68], who have indicated that cell division can continue without exogenous cytokinin due to the tissue being able to produce endogenous cytokinin in an adequate amount. Correspondingly, the explant itself can provide the metabolites and cell division factors that are essential for the initiation, organization, and development of the buds [69]. Meanwhile, exogenous PGRs are also important as it influences most metabolic processes such as initiation of shoots and roots, cell division and differentiation of plantlet and callus [70]. The type and concentration of PGRs in a tissue culture largely affect the efficiency, rate of multiplication and elongation of the shoot and root formation as well as photomorphogenesis of plantlets.

The difference between the results might be due to different levels of endogenous PGRs within the explants and exogenous factors such as light intensity, temperature, and relative humidity within the culture room [71]. The growth and development of in vitro plantlets were also affected by the concentration of carbon dioxide (CO_2_) and ethylene (C_2_H_4_) [72]. In addition, different varieties, ages and genotypes of explants behave differently during in vitro culture [73,74].

In general, the organogenic ability of an explant is highly dependent on totipotency and plasticity. In this research, one significant finding was that the primary shoots in medium supplemented with exogenous cytokinin were directly induced axillary buds from the nodal segment of *P. niruri* explant, without the intermediate callus phase. This was possible because of the totipotent and plasticity nature of *P. niruri* explant, which initiates the cell division hence regenerates the primary shoot.

Root induction and growth at the base of in vitro grown shoot are an essential and vital stage in plant micropropagation establishment of plantlet before transferring to the field. For this purpose, the application of auxin as a vital hormone for rooting initiation is necessary. Root production of *P. niruri* was successfully achieved after four weeks of culture.

Of the auxins tested, 2.5 µM of IBA was the best for proper rooting, in which it produced the highest number of roots compared to other treatments. Tanimoto [75] has shown that IBA is the most efficient auxin for olive rhizogenesis compared to NAA. The explanation for the difference in root-inducing potential could be due to the slow release of IAA from IBA and the release of IBA through conjugate hydrolysis [76]. After absorption, IBA could be conjugated with amino acids, or IBA transformed into IAA [77]. In serving as an auxin source during the later stage of rooting, these IBA conjugates were stated to be superior [78]. Additionally, IAA uptake was about four times lower than IBA uptake [76]. These findings agree with the study conducted by Kalidass and Mohan [64], who proved that IBA in the range of concentrations 2.0–3.0 µM generated the most significant number of roots in *Phyllanthus urinaria* as compared to IAA and NAA treatments. In contrast, working with the same variety, Kalidass and Mohan [79] recorded that NAA with the concentration of 1.25–5.0 µM gave the highest number of roots per plantlet. The inferior result of NAA on the number of roots might be because NAA is more persistent than IBA. It stays present in the tissue in its pure state and can prevent root meristemoids from developing further [80].

The highest length of the root was found in media supplemented with 2.5 µM of IBA with 1.29 cm while retaining no significant difference with IBA 1.25 µM (1.07 cm) and NAA 2.5 µM (1.22 cm). Another study was performed on *Olea europaea* L. cv. “Moraiolo” by Ansar et al. [81] produced a similar result whereby they found that IBA at 1.5 mg/L concentration produced the highest root length (4.95 cm) compared to treatment with a lower concentration of IBA and all other NAA treatments. They also observed that the root produced on IBA were longer with a better quality of shoots while NAA developed poor response with leaf abscission and necrotic leaves. Cell elongation requires a sequential adjustment in enzymes’ amount and activity; hence, auxin activates the enzymes involved in cell enlargement. Wada et al. [82] proved that IBA boosts root length by affecting the synthesis of enzymes involved in cell enlargement. Ludwig-Müller [83] revealed that many factors might be responsible for the excellent effects of IBA on root elongation compared to NAA, such as its preferential uptake, subsequent gene activation, transport, and mobilization. It was observed that the type and concentration of auxin strongly affected the rooting response at the rooting phase. Kollmeier et al. [84] reported that the root elongation stage is susceptible to auxin concentration which high concentrations may inhibit the root development. The supra-optimal concentration of auxins probably inhibits root elongation by enhancing ethylene biosynthesis.

Acclimatization is an essential step in plant micropropagation because it ensures that plantlets grown in vitro adapt to their natural environment when transplanted into nature. These plantlets must adjust to environmental conditions, including humidity, temperature, photoperiod, and pH gradually. As per Kirdmanee et al. [85], the number of plantlets successfully transferred to glasshouse or field conditions can be used to assess in vitro propagation success.

The current study found that the mixture of coco peat and peat moss enhanced the plantlets’ quality, allowing them to withstand the harsh external climate. The results were in accordance with that of Manjusha and Sathyanarayana [86], who also stated the highest percentage of stevia plantlet was reported with cocopeat media. High establishment success in peat might be attributed to better aeration, high water holding capacity, and moderate pH [87].

After 4 weeks under ex vitro conditions, the plantlet survival rate was over 80%, demonstrating that this procedure and protocol can be utilized as an efficient method for plant acclimatization. Furthermore, the ex vitro established plantlets displayed normal growth characteristics with no noticeable abnormalities. Hence, potting media treatment of coco peat + peat moss was optimal for acclimatization of *P. niruri,* which produced the highest percentage of plantlet survival.

## 4. Materials and Methods

### 4.1. Plant Materials and Maintenance

*P. niruri* plant was collected from the UPM compound and garden. A voucher specimen of P. niruri was deposited at the Institute of Bioscience, UPM, to identify and confirm plant species and given the voucher number SK 3356/18.

### 4.2. Surface Sterilization of Explants

The explants were obtained by excising the stem nodes of P. niruri. The stem nodes were cut into 1–1.5 cm long and washed with detergent for 30 min under running tap water. Thereafter, the surface sterilization procedure was conducted under a sterilized condition in a laminar flow hood. The explants were sterilized using 70% ethanol for 30 s then washed with sterile distilled water at least three times. From here on, the explants were submerged into different chemical sterilants (Clorox; 10, 20 and 30% and nano silver; 10, 20 and 30 ppm) with an addition of 2–3 drops of Tween 20 surfactant and shaken for 30 min. After that, all the treatment solutions were discarded, and the explants were rinsed at least three times with sterile distilled water.

The parameter taken for this experiment was the percentage of contamination by counting the number of contaminated explants in each flask at the end of the second week after cultivation. The flask containing cultured explants was incubated for two weeks at a temperature of 25 ± 3 °C under 16 h light and 8 h dark of photoperiod alongside white fluorescent light radiation of 45 µmol/m²/s in the incubation room.

From the counting, the percentage of contamination was measured according to the formula:

$$\frac{\mathrm{Number}\;\mathrm{of}\;\mathrm{contaminated}\;\mathrm{explants}\;}{\mathrm{Number}\;\mathrm{of}\;\mathrm{total}\;\mathrm{cultured}\;\mathrm{explant}}\;\times 100\;(\%)$$

### 4.3. Basal Media and Its Strength

Two types of basal media prepared using media formulation were explained by Murashige and Skoog (Sigma-Aldrich, St. Louis, MI, USA) containing iron chelated to the disodium salt of EDTA and Gamborg et al. (Duchefa Biochemie, The Netherlands) which containing iron chelated to the mono-sodium salt of EDTA. Briefly, the macronutrients, micronutrients, and vitamins solution was mixed with 0.1 g/L of myo-inositol (Duchefa Biochemie, The Netherlands) and 30 g/L of sucrose (Duchefa Biochemie, The Netherlands). The medium’s pH was adjusted to pH 5.6–5.8 by adding 1 M of either sodium hydroxide (NaOH) and hydrochloric acid (HCl). A total of 3 g/L of Gelrite™ (Duchefa Biochemie, The Netherlands) was added and stirred until completely dissolved. The solution was heated in the microwave before being poured into vials. All the labeled vials were then placed in the autoclave to be sterilized at 121 °C and 1.05 kg/cm² for 20 min The strength of basal media (half, full- and double-strength) was manipulated by either reducing or amplifying the macronutrients, micronutrients, and vitamins. MS with full strength served as a control treatment in this experiment. The data were taken on the number of shoots, length of the shoot (cm), and the number of leaves was recorded after four weeks of incubation.

### 4.4. Plant Growth Regulators and Its Concentrations

The best basal medium and its strength from the previous experiment were prepared and supplemented with different cytokinin; 6-benzylaminopurine (BAP), kinetin (Kn), Zeatin (Zn), and 2-isopentenyl adenine (2iP) (Duchefa Biochemie, Netherlands) at different concentrations of 2.5, 5, 7.5, 10.0 µM. The control treatment was devoid of any plant growth regulators. The cultures were maintained for four weeks of incubation. The parameters taken for this experiment were the number of shoots, length of the shoot (cm) and the number of leaves. All the data collection was carried out after four weeks of culturing.

### 4.5. In Vitro Rooting and Acclimatization

The explants from the previous experiment were subcultured to a fresh MS media containing different types of auxin at different concentrations. The basal media was supplemented with different auxin; indole-3-butyric acid (IBA), 1-naphthalene acetic acid (NAA), and indole-3-acetic acid (IAA) at different concentrations of 1.25, 2.5, and 5 µM. Media without any auxin serve as control treatment. The parameters taken for this experiment were the number of roots and length of root (cm). All the data collection was carried out after four weeks of culturing. Root induction and development parameters were taken on week four of inoculation.

Rooted explants were removed and rinsed gently with distilled water to remove any agar trace before transferring to pot under ex vitro condition in potting media containing peat moss (Hup Nong Agriculture Sdn. Bhd.) and cocopeat (Cofibers Sdn. Bhd.) at ration 1:1. Any trace of agar might encourage fungal and bacterial infections. The plantlets were kept in a shaded area and watered daily for four weeks. The plastic cover was gradually removed after the first week, and plantlet were exposed to 70% humidity and temperature of 28 ± 4 °C.

The percentage of survival was calculated using the formula as below:

$$\mathrm{Percentage}\;\mathrm{of}\;\mathrm{survival}=\frac{\mathrm{No}.\;\mathrm{of}\;\mathrm{surviving}\;\mathrm{plantlets}}{\mathrm{Total}\;\mathrm{number}\;\mathrm{of}\;\mathrm{plantlets}\;\mathrm{transferred}}\;\times \;100\%$$

### 4.6. Experimental Design and Data Analysis

All experiments were conducted in a completely randomized design and were repeated twice. Each treatment consisted of three replicates. Mean values of various treatments were subjected to analysis of variance (ANOVA), and the significant difference was separated using Least Significant Difference (LSD). SAS version 9.4 was used to determine the significance at *p* < 0.05.

## 5. Conclusions

This current study suggested an efficient protocol for in vitro propagation of *P. niruri*. The establishment of aseptic culture was achieved using 30% of Clorox^®^ as a sterilizing agent to diminish microbial contaminations. Various plant growth factors which including basal medium and plant growth regulators were studied to find the best formulation for the growth and development of *P. niruri*. According to the findings, full-strength MS basal medium with no cytokinin content (control) treatment produced the best results with a high number of shoots, length of shoot and number of leaves. The root induction of *P. niruri* explant has been influenced by different types and concentrations of auxins, as the result showed the media supplemented with 2.5 µM of IBA exhibited the best result, which produced 17.92 roots and 1.29 cm length of roots after four weeks of incubation.

Besides that, ex vitro establishment was achieved with an 88% of survival rate of *P. niruri* plantlet in a media mixture of coco peat and peat moss at a ratio of 1:1. This study has effectively developed a protocol for propagating *P. niruri* through plant tissue culture. This research can therefore contribute to the production of *P. niruri* raw materials to meet the requirements of the herbal industry. Originally, it was assumed that all plants regenerated from cell or tissue culture would have genetic materials identical to the parent plant. Despite this, phenotypic variation must be common between regenerated plants. Thus, further studies on DNAs of regenerated plantlets can be conducted to determine its somaclonal variation level when compared to the mother plant.

## Figures and Tables

**Table 1:** Effect of different chemical sterilants and concentrations towards the percentage of contamination (%) of *P. niruri* on week 2 of incubation.

Treatment/Chemical Sterilants	Concentration	Percentage of Contamination (%)
Clorox^®^ (%)	10	10.22 ± 2.28 ab
	20	6.67 ± 0.7 bc
	30	4.44 ± 1.21 c
Nano Silver (ppm)	10	14.22 ± 1.13 a
	20	12.44 ± 1.13 a
	30	12.00 ± 2.28 a

Means followed by a similar alphabet within the column are not significantly different at *p* ≤ 0.05 using the LSD test.

**Table 2:** Effect of different basal media and its strength towards the number of shoots, length of the shoot (cm), and number of leaves of *P. niruri*.

Treatment	Number of Shoots	Length of Shoot (cm)	Number of Leaves
MSH	2.00 ± 0.57 bc	2.79 ± 0.14 bc	23.11 ± 0.94 a
MSF	2.33 ± 0.33 ab	3.11 ± 0.43 b	27.91 ± 0.90 a
MSD	0.67 ± 0.33 c	1.5 ± 0.23 c	4.88 ± 0.89 b
B5H	3.67 ± 0.33 a	3.52 ± 0.12 ab	24.25 ± 0.47 a
B5F	3.00 ± 0.57 ab	4.84 ± 0.33 a	35.94 ± 1.81 a
B5D	1.67 ± 0.66 bc	3.58 ± 0.69 ab	22.83 ± 0.92 a

Means followed by the similar alphabet within the column are not significantly different at *p* ≤ 0.05 using Least Significant Difference (LSD) test. MS: Murashige and Skoog, B5: Gamborg B5, H: Half-strength, F: Full-strength, and D: Double-strength.

**Table 3:** Effect of different cytokinin and its concentrations towards the number of shoots, length of the shoot (cm), and number of leaves of *P. niruri*.

Cytokinin (µM)	Number of Shoots	Length of Shoot (cm)	Number of Leaves
Control	5.0 ± 0.50 ab	3.68 ± 0.60 ab	27.33 ± 1.15 a
BAP 2.5	4.45 ± 0.96 abc	3.09 ± 0.45 bcd	20.89 ± 1.29 abcd
BAP 5.0	5.33 ± 0.57 a	2.5 ± 0.51 bcde	24.11 ± 2.00 abc
BAP 7.5	4.45 ± 0.77 abc	2.47 ± 0.44 bcde	23.22 ± 1.83 abc
BAP 10.0	2.55 ± 0.61 e	1.67 ± 0.15 e	16.00 ± 1.83 bcd
Kn 2.5	5.33 ± 0.77 a	3.38 ± 0.42 abc	25.33 ± 0.62 ab
Kn 5.0	4.34 ± 0.33 abcd	4.31 ± 0.89 a	22.45 ± 0.49 abc
Kn 7.5	4.1 1± 0.29 abcd	2.38 ± 0.30 cde	18.67 ± 2.05 abcd
Kn 10.0	3.67 ± 0.50 bcde	2.73 ± 0.58 bcde	18.33 ± 1.50 abcd
Zn 2.5	3.55 ± 0.39 bcde	2.52 ± 0.07 bcde	15.46 ± 1.61 cd
Zn 5.0	2.89 ± 0.11 de	2.01 ± 0.09 de	17.00 ± 2.71 bcd
Zn 7.5	3.22 ± 0.29 cde	1.99 ± 0.06 de	14.67 ± 1.64 cd
Zn 10.0	4.22 ± 0.11 abcd	2.56 ± 0.09 bcde	2.22 ± 1.44 abc
2iP 2.5	3.89 ± 0.72 abcde	2.71± 0.36 bcde	15.22 ± 1.36 cd
2iP 5.0	3.0 ± 0.33 cde	1.63 ± 0.20 e	12.33 ± 0.38 d
2iP 7.5	3.45 ± 0.22 cde	2.43 ± 0.56 cde	16.00 ± 2.54 bcd
2iP 10.0	4.00 ± 0.57 abcde	2.47 ± 0.16 bcde	15.89 ± 1.89 bcd

Means followed by the similar letter within the column are not significantly different at *p* ≤ 0.05 using Least Significant Difference (LSD) test. BAP: 6-benzylaminopurine, Kn: kinetin, Zn: Zeatin, 2iP: 2-isopentenyl adenine.

**Table 4:** Effect of different auxins and their concentrations towards the number of roots and length of root (cm) of *P. niruri*.

Auxin (µM)	Number of Roots	Length of Root (cm)
Control	1.0 ± 0.40 h	0.13 ± 0.03 f
IBA (1.25)	9.4 ± 1.11 cd	1.07 ± 0.03 abc
IBA (2.5)	17.92 ± 1.38 a	1.29 ± 0.11 a
IBA (5)	10.53 ± 0.67 bc	0.79 ± 0.19 cde
IAA (1.25)	7.27 ± 0.40 de	0.97 ± 0.09 bcd
IAA (2.5)	3.47 ± 0.24 g	0.85 ± 0.05 cde
IAA (5)	4.87 ± 0.33 fg	0.86 ± 0.06 cde
NAA (1.25)	6.93 ± 0.94 ef	0.65 ± 0.11 e
NAA (2.5)	12.47 ± 0.88 b	1.22 ± 0.08 ab
NAA (5)	11.87 ± 0.43 b	0.77 ± 0.06 de

Means followed by the similar letter within the column are not significantly different at *p* ≤ 0.05 using Least Significant Difference (LSD) test. IBA: indole-3-butyric acid, NAA: 1-naphthalene acetic acid, IAA: indole-3-acetic acid.

## Data Availability

The data presented in this study are available in the article.
